# Pattern recognition of the fluid flow in a 3D domain by combination of Lattice Boltzmann and ANFIS methods

**DOI:** 10.1038/s41598-020-72926-3

**Published:** 2020-09-28

**Authors:** Meisam Babanezhad, Ali Taghvaie Nakhjiri, Azam Marjani, Saeed Shirazian

**Affiliations:** 1 Institute of Research and Development, Duy Tan University, Da Nang, 550000 Vietnam; 2 Faculty of Electrical – Electronic Engineering, Duy Tan University, Da Nang, 550000 Vietnam; 3 Department of Petroleum and Chemical Engineering, Science and Research Branch, Islamic Azad University, Tehran, Iran; 4 Department for Management of Science and Technology Development, Ton Duc Thang University, Ho Chi Minh City, Vietnam; 5 Faculty of Applied Sciences, Ton Duc Thang University, Ho Chi Minh City, Vietnam; 6 Department of Chemical Sciences, Bernal Institute, University of Limerick, Limerick, Ireland; 7 Laboratory of Computational Modeling of Drugs, South Ural State University, 76 Lenin Prospekt, Chelyabinsk, Russia 454080

**Keywords:** Chemical engineering, Mechanical engineering, Engineering, Mathematics and computing

## Abstract

Many numerical methods have been used to simulate the fluid flow pattern in different industrial devices. However, they are limited with modeling of complex geometries, numerical stability and expensive computational time for computing, and large hard drive. The evolution of artificial intelligence (AI) methods in learning large datasets with massive inputs and outputs of CFD results enables us to present completely artificial CFD results without existing numerical method problems. As AI methods can not feel barriers in numerical methods, they can be used as an assistance tool beside numerical methods to predict the process in complex geometries and unstable numerical regions within the short computational time. In this study, we use an adaptive neuro-fuzzy inference system (ANFIS) in the prediction of fluid flow pattern recognition in the 3D cavity. This prediction overview can reduce the computational time for visualization of fluid in the 3D domain. The method of ANFIS is used to predict the flow in the cavity and illustrates some artificial cavities for a different time. This method is also compared with the genetic algorithm fuzzy inference system (GAFIS) method for the assessment of numerical accuracy and prediction capability. The result shows that the ANFIS method is very successful in the estimation of flow compared with the GAFIS method. However, the GAFIS can provide faster training and prediction platform compared with the ANFIS method.

## Introduction

Artificial intelligence (AI) has been frequently used in the prediction of physical and industrial processes^1–4^. They are also used as an assistance tool besides exact studies, either numerical or experimental, during optimization of processes, and they can mimic and then provide mathematical descriptions for processes^5–8^. Recently, AI has been combined with computational fluid dynamics to simulate the fluid flow pattern in different geometries, such as square cavity and the cylindrical bubble column reactors^9–11^. In this combination, the AI learns the process from the computational fluid dynamics (CFD) data set, specifically from each CFD node in the domain, and then represents the new data set for different conditions. The flow pattern in this condition is also based on the CFD data and is completely independent of boundary conditions, mesh sensitivity, and stability of numerical methods^12–15^. The AI shows the evolution of the flow pattern between the range of exact models, which is helpful to avoid exact modeling repetition with expensive computational expenses in the optimization process.

In the multiphase flow application, Pourtousi et al.^16^ employed the ANFIS method to learn CFD data from different heights of a bubble column reactor and predicted the new data set of the flow pattern for different heights and sparger (gas distributor) specifications. They also used this method to learn CFD data about formation, detachment and to raise the bubble at few CFD time steps, and after learning data, they predicted the interface between continuous and dispersed phase for the very small time step. Pourtousi et al.^17^ showed that the prediction of the interface between dispersed and continuous phase is not very accurate with the combination of ANFIS and CFD, and it requires a huge number of rules.

There are also several tuning parameters to accurately predict the shape of the pattern in the AI^18^. For instance, in the ANFIS technique, number of rules and membership functions can be changed for each input and results in improvement of the pattern^6,19^.

The selection of different datasets during the learning process enables us for better pattern recognition. One of the main advantages of this accurate fluid flow pattern prediction is the CFD method does not require to save each time step and then store the data. In this case, AI plays a role as an assistance tool to provide non-existing data, which sometimes needs large computational time and hard drive for storing data. This technique can be replaced with computational fluid dynamics, and it enables us to avoid storing data for a very small time step and replace smart modeling instead of CFD modeling.

Recently there are several machine learning (ML) tools that have been developed to predict the pattern of flow in a domain. They showed that the number of input parameters and the number of membership functions could significantly impact on accurate prediction of flow pattern. However, the selection of each model based on the learning time has not been fully considered. Additionally, the prediction of more futures in the pattern has not been fully investigated. In this work, we consider the prediction of flow pattern in the domain and represent new features of flow characteristics based on the predictions ability.

We use different patterns of fluid flow for different time steps as a data set, and with the ANFIS method, all time steps are learned. After training all patterns of fluid, the AI predicts missing times with the CFD method, which has not been used in the training method. We also compare prediction results with the existing model in literature called the genetic algorithm fuzzy inference system (GAFIS) to evaluate the capability of models in predicting flow patterns. For the first time, we present a new mathematical correlation based on AI for the flow pattern in the cavity domain. This correlation can represent the local values for the fluid flow when there is a shear flow.

## Method

In this study, to build-up a large dataset for the fluid flow, we simulate the 3D cavity by the Lattice Boltzmann method (LBM). This dataset enables us to study the ability of ML prediction process in the simulation of fluid flow pattern recognition in a simple fluid problem. The AI is used to get several slices for various simulation time at the center of the cavity, in x–y coordinate. Then it tries to learn the process and predict many simulation times that are not simulated by the CFD method or saved on the computer. This ability enables us to visualize the fluid pattern in a short period of time.

### Lattice Boltzmann Method (LBM)

For simulation of single-phase fluid flow in the 3D cavity, we use the lattice Boltzmann method, and the collision term is computed based on Bhatnagar-Gross-Krook (BGK). The model of D3Q19 is used to present the location of LB points. The LB equation, which represents the streaming and collision part, is written as:

1 $$f_{i} \left( {\vec{x} + c\vec{e}_{i} \Delta x,t + \Delta t} \right) - f_{i} \left( {\vec{x},t} \right) = \frac{1}{\tau }\left( {f_{i}^{ eq} \left( {\vec{x},t} \right) - f_{i} \left( {\vec{x},t} \right)} \right) + \Delta tF_{i}$$

where $f_{i}$ is the density distribution and $f_{i}^{{{ }eq}}$ is the equilibrium distribution for particles in the domain. $c_{i} = ce_{i}$ is the discrete velocity in the domain of cavity, while $e_{i}$ presents unit lattice velocity. $F$ is also the external force in the direction of $i$.

The equilibrium distribution ($f_{i}^{eq}$) with different weight factors ($w$) describes as:

2 $$f_{i}^{eq} = w_{i} \rho \left\{ {1 + \frac{{ _{{c_{i} u^{eq} }} }}{{C_{S}^{2} }} + \frac{{(c_{i} u^{eq} )^{2} }}{{2C_{S}^{2} }} - \frac{{u^{eq2} }}{{2C_{S}^{2} }}} \right\}$$

The weight factors are as *w*_*0*_ = 1/3, *w*_*1−6*_ = 1/18 and *w*_*5−9*_ = 1/36. The macroscopic density and velocity of fluid can be calculated based on:

3 $$\rho = \sum\limits_{i} {f_{i} }$$

4 $$\rho \vec{u} = \sum\limits_{i} {\vec{e}_{i} } f_{i}$$

$\rho$ and $u$ present the density and velocity of fluid flow, respectively.

### ANFIS

ANFIS is a fuzzy implication structure that precisely forecasts the manners of nonlinear and complex systems^20,21^. Three various sorts of fuzzy reasoning are present, which Sugeno and Takagi suggested if–then rules applied in the ANFIS framework^22^. Herein x coordination (x), y coordination (y), and time (t) are engaged for the achievement of fluid velocity in place of output. The function of the *i*th rule is written as:

5 $$w_{i} = \mu_{Ai} \left( x \right) \mu_{Bi} \left( y \right)\mu_{ci} \left( t \right)$$

where *w*_*i*_ is the signal coming out of the second layer's node and *μ*_*Ai*_, *μ*_*Bi*_ and *μ*_*Ci*_ are received signals from implemented MFs on inputs, x coordination (x), y coordination (y) and time (t), to the second layer's node. More details on ANFIS can be found elsewhere^22^.

## Results and discussion

The analysis of liquid flow pattern by CFD methods in 3D geometries requires high computational time. Saving all fluid properties in the three dimensions for each time step is time-consuming and needs a very large hard drive for storing data. In this study, we specifically, simulate the 3D cavity and save data at the center of the domain for several time steps. Then we use some data in training ANFIS for prediction of the liquid pattern. During learning data, we examine different combinations of membership functions, rules, and the number of inputs to evaluate the best condition for the prediction of flow patterns with AI. After learning all data, we call ANFIS function to generate the liquid flow pattern for different time steps, and we compare them with some of the CFD data that has not been presented in the training process. This procedure enables us to facilitate fast visualization of the data in a short computational time.

**Figure 1:**
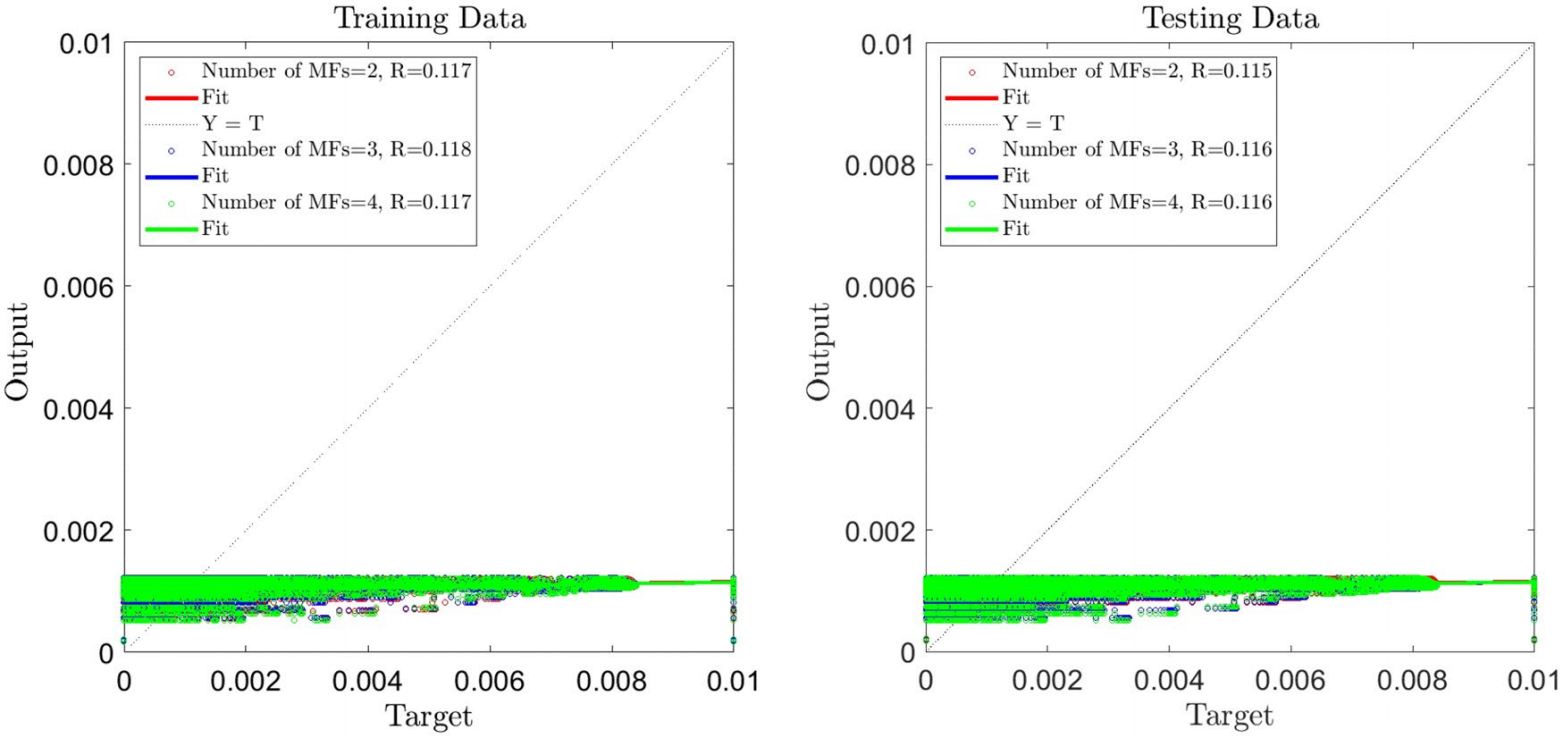
ANFIS training and testing processes, one input, number of MFs = 2, 3, 4.

The prediction of flow pattern recognition in the domain requires the high accuracy of the learning process in artificial intelligence algorithms. This accuracy called "intelligence of the method" can be achieved with all tuning parameters in the AI and way of processing and training data. In this study, to achieve the intelligence of the method, we start training with different numbers of inputs and outputs. At first, we train data with one input and output and observe the accuracy of the method. The results show that the accuracy of the method is very low when only one input is used in the training process. This accuracy can not increase by increasing the number of rules or the number of membership functions. To achieve better accuracy of the method, we can increase the number of inputs. Increasing the number of inputs causes an increment in the number of neural in the system. For clustering data in the ANFIS method, we select grid partition clustering and also select generalized bell-shaped membership function (Gbellmf) as a type of fuzzy membership functions (MFs). The percentage of data utilized for training is %60, the maximum iteration in ANFIS is 500, and the number of data is considered 65,000. With the above parameters and considering x coordinate as the first input and different number of MFs, the ANFIS learning is implemented. Figure 1 shows the regression (R) about 0.117 for the training, and 0.116 for the testing. This value of R shows %11 of ANFIS intelligence for reaching a high ANFIS intelligence, we consider y coordinate as the second input and repeat the ANFIS training/testing when the number of MFs = 2,3 and 4.

**Figure 2:**
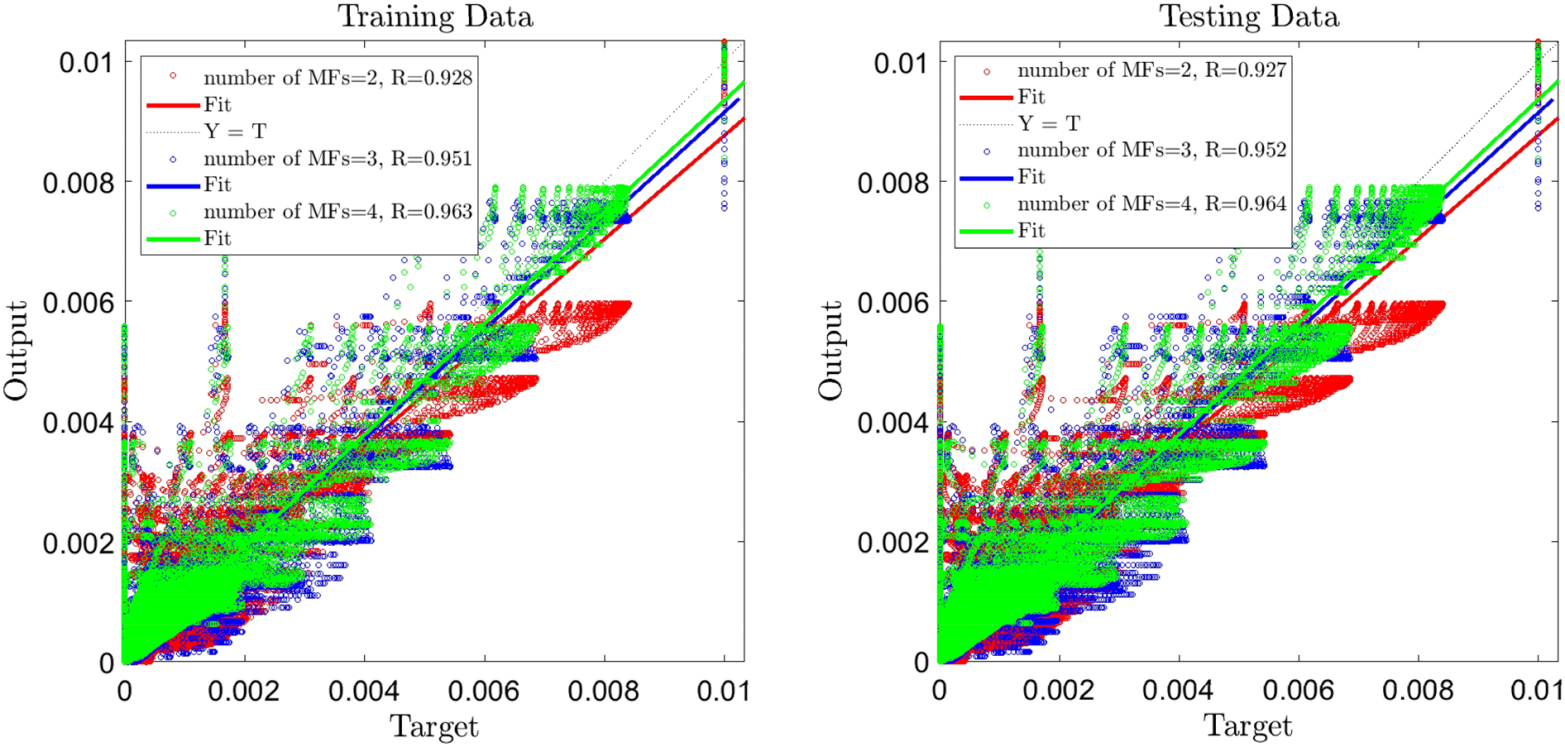
ANFIS training and testing processes, two inputs, number of MFs = 2, 3, 4.

**Figure 3:**
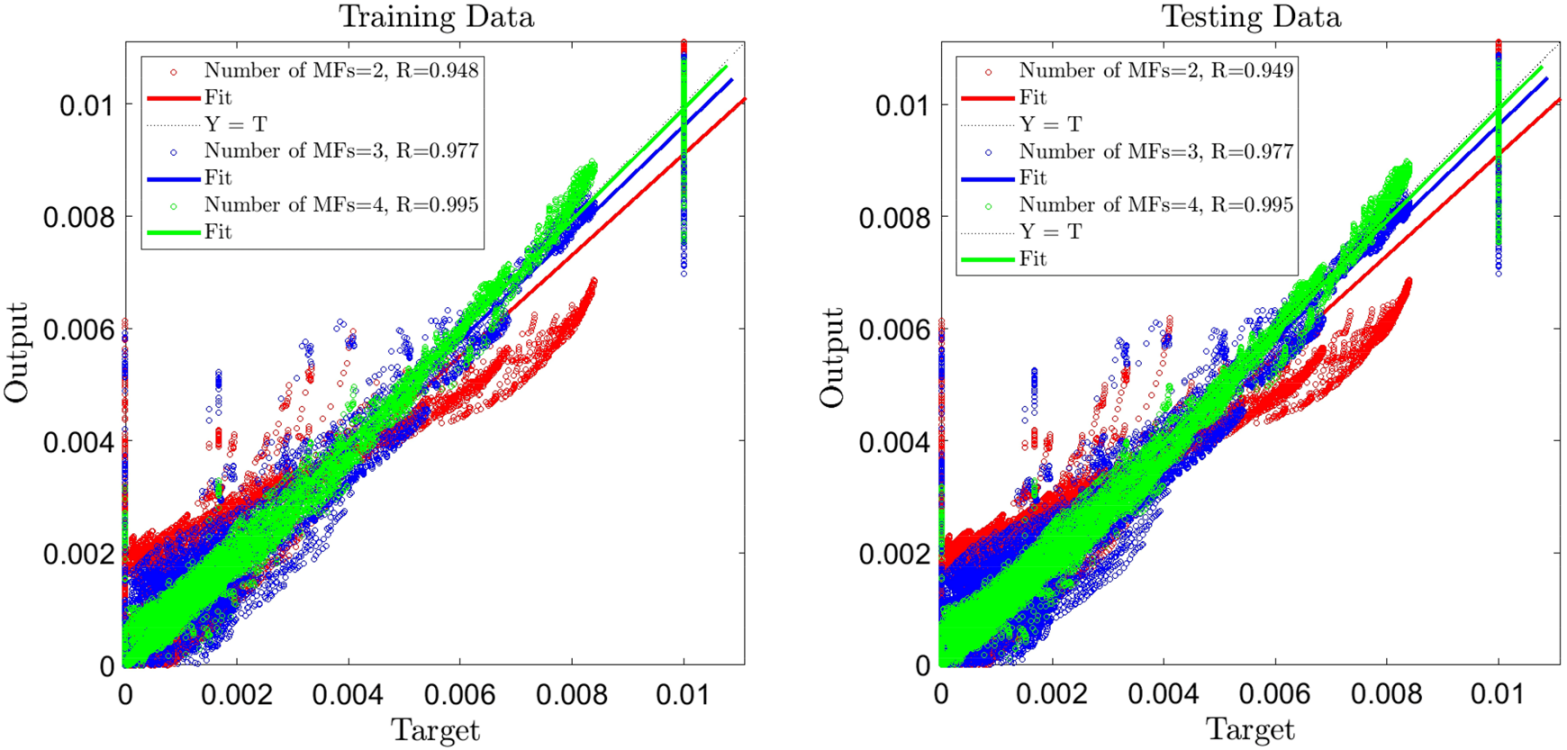
ANFIS training and testing processes, three inputs, number of MFs = 2, 3, 4.

Results depicted in Fig. 2 reveal that the amount of R is drastically risen up, and when the number of MFs = 2, the value of R for training/testing is about 0.92. Changes in the number of MFs from 2 to 4 indicated an increase in the amount of R from 0.92 to 0.96, which had a great influence over achieving %96 of ANFIS intelligence which is depicted in Fig. 3.

To reach a high percentage of ANFIS intelligence, we add time as the third input, and the learning step for the number of MFs = 2, 3, and 4 are done. By comparing R when the number of inputs is three and two, which indicates an increase in R in the testing and training, particularly when the number MFs = 4, percentage of ANFIS intelligence is %99.5, which is a significant achievement in the ANFIS intelligence.

**Figure 4:**
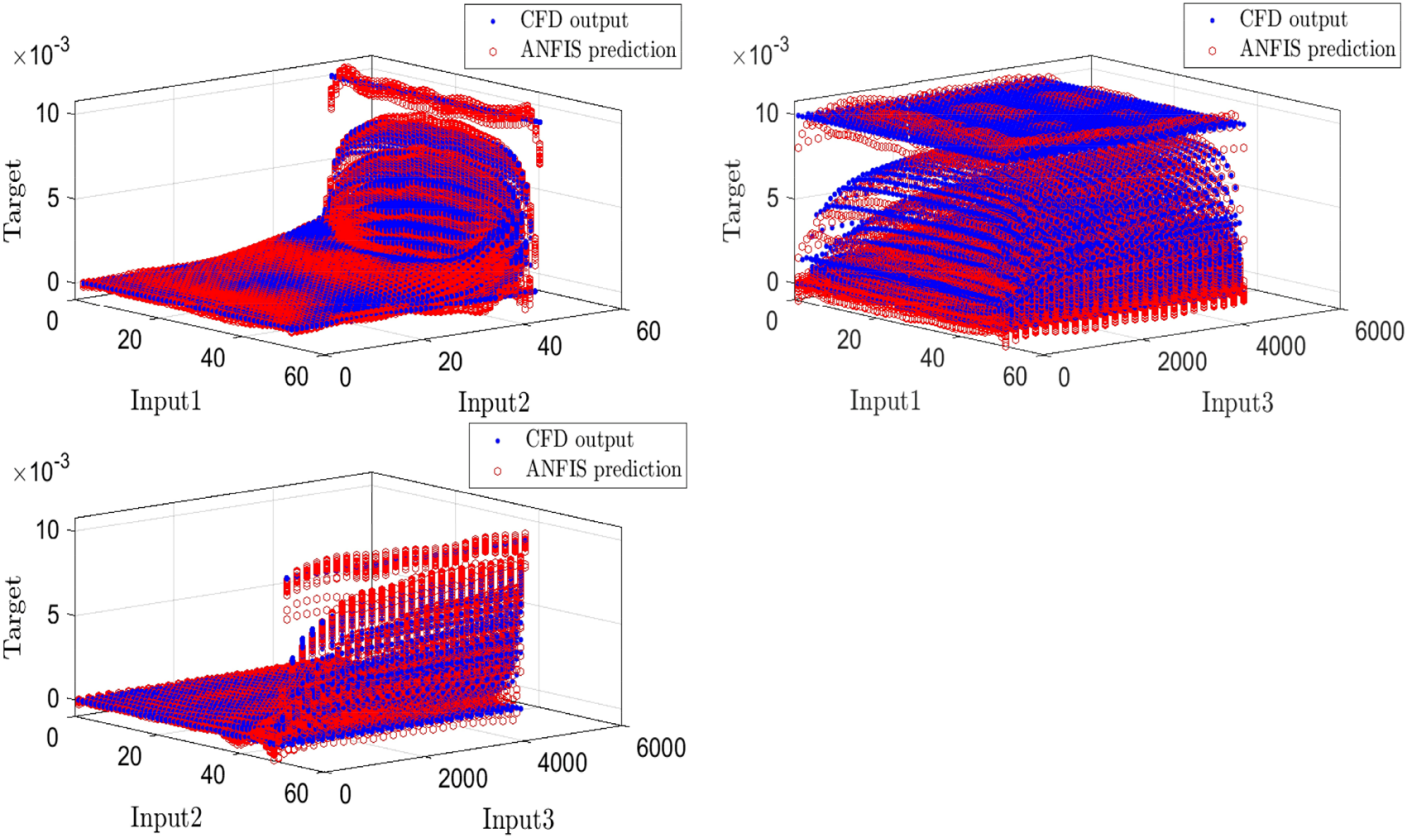
ANFIS prediction validation by the CFD outputs, three inputs, number of MFs = 4.

**Figure 5:**
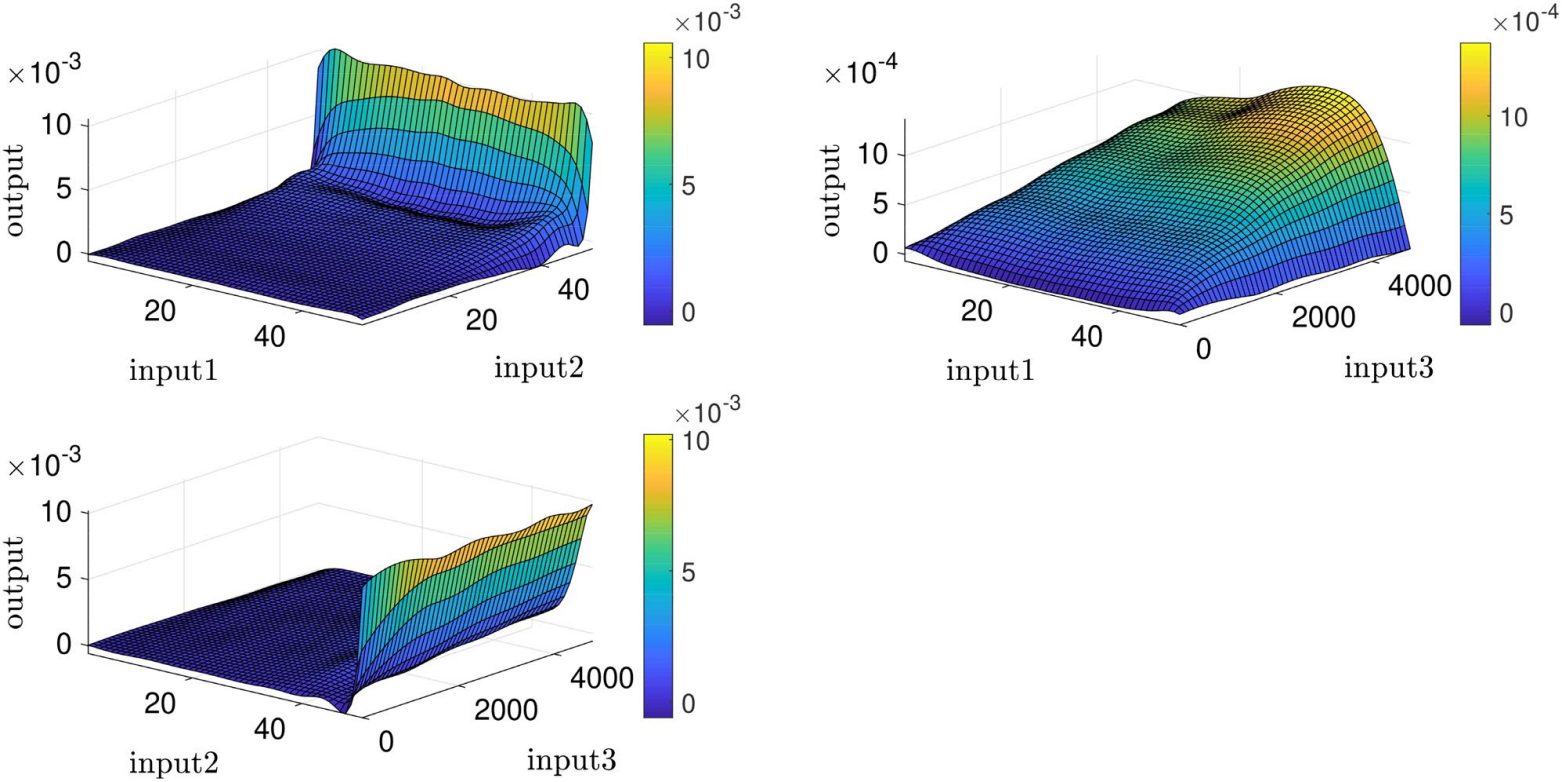
ANFIS prediction surfaces, three inputs, number of MFs = 4.

According to Fig. 4, ANFIS prediction points have good adaptation with the CFD points; eventually, we predict surfaces that indicate velocity as the ANFIS output based on different inputs. By using predicted surfaces, there is a suitable capability to achieve more points in the cavity, which most of them have not been present in the ANFIS learning processes (see Fig. 5).

**Figure 6:**
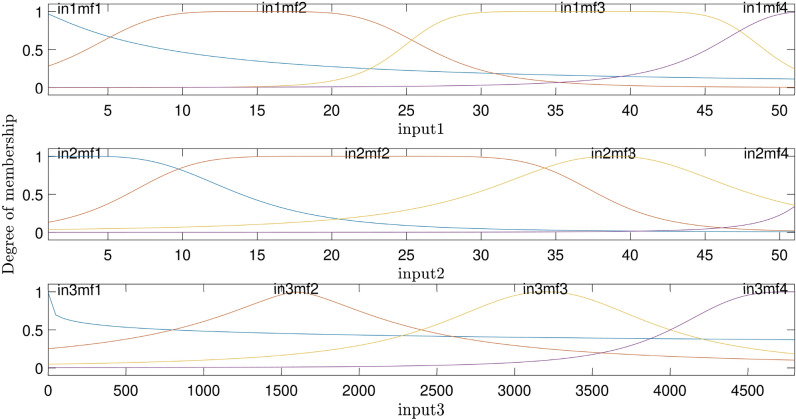
Degree of membership function, three inputs, number of MFs = 4.

**Figure 7:**
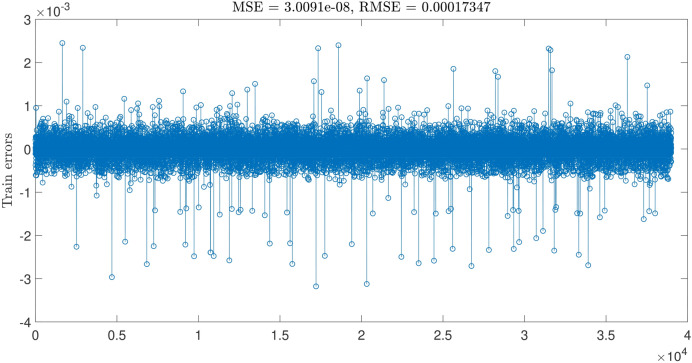
ANFIS training errors, three inputs, number of MFs = 4.

**Figure 8:**
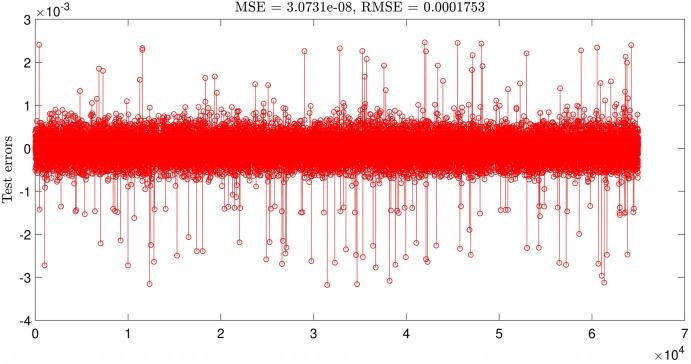
ANFIS testing errors, three inputs, number of MFs = 4.

**Figure 9:**
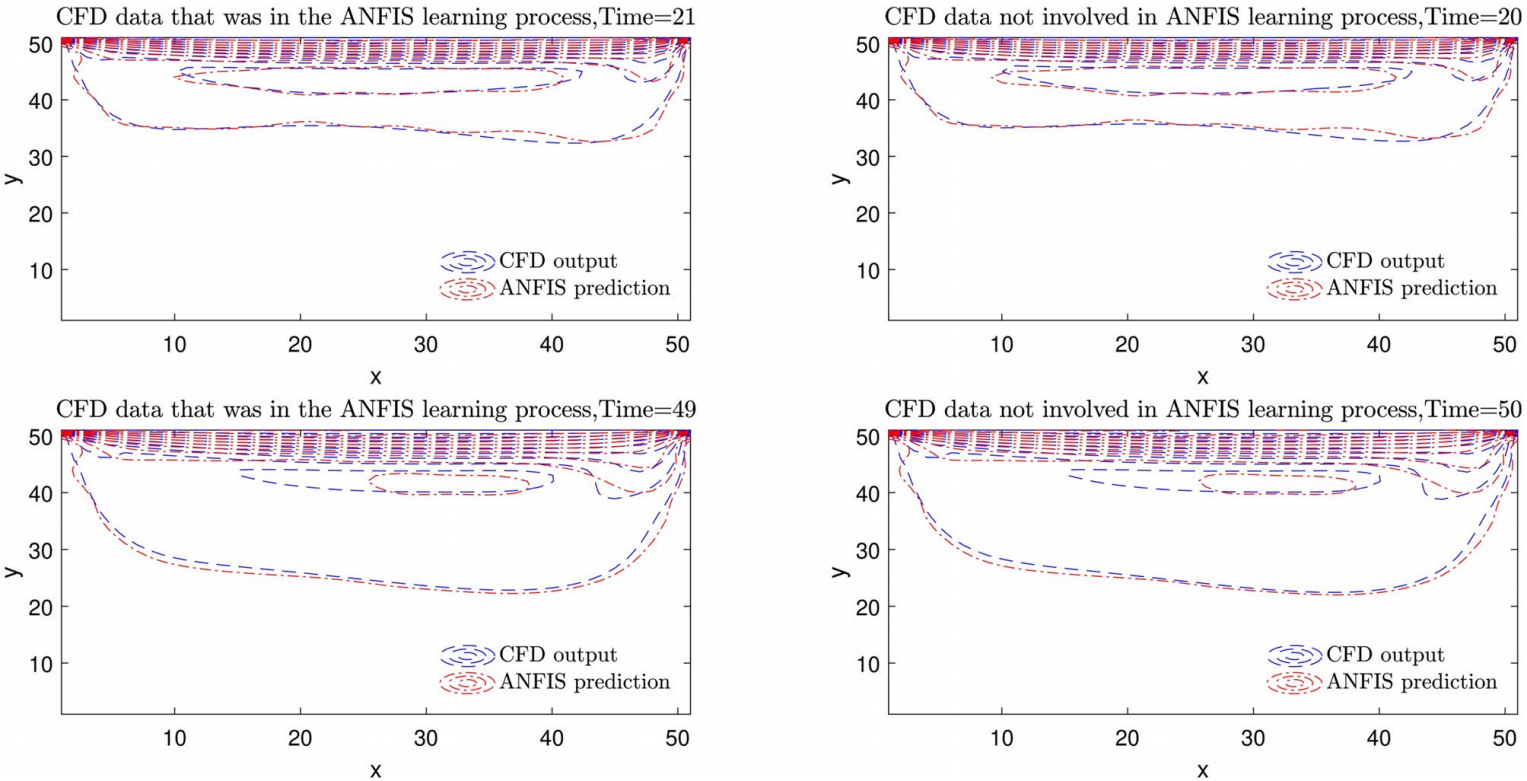
Velocity prediction pattern for absent and present times in learning processes by the obtained ANFIS intelligence when the number of inputs is three, and the number of MFs is 4.

The highest intelligence is achieved when the number of inputs equals three and the number of MFs = 4. The degree of MFs is illustrated in Fig. 6; furthermore, Figs. 7 and 8 indicate MSE error and RMSE error for the training and testing processes in the highest level of ANFIS intelligence. In the following section, the velocity prediction patterns are depicted for the times that its data are present in the ANFIS learning processes, the velocity prediction patterns are depicted on the left side of Fig. 9, also the velocity prediction patterns for the times that are absent in the learning processes are depicted on the right side of Fig. 9.

**Figure 10:**
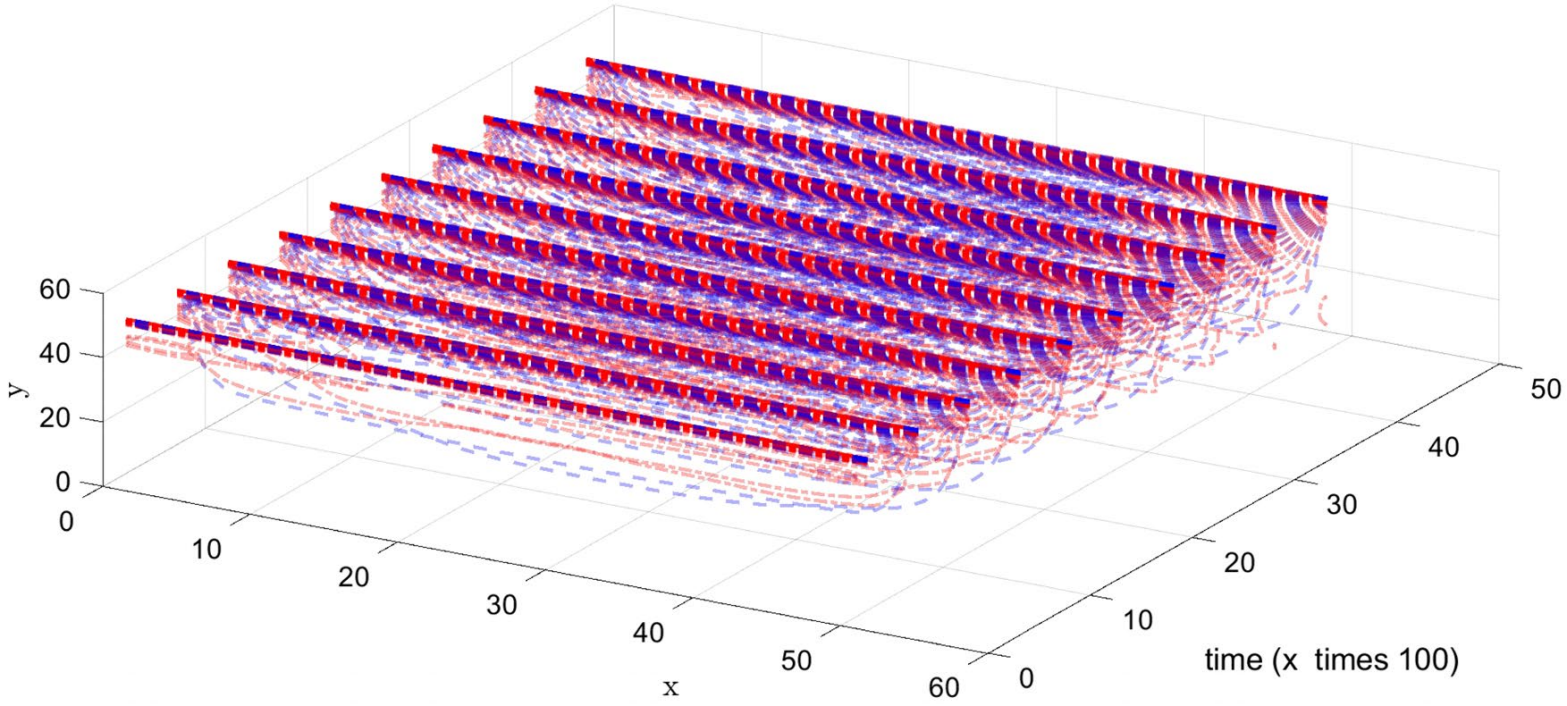
Velocity prediction 3D pattern, three inputs, number of MFs = 4.

**Figure 11:**
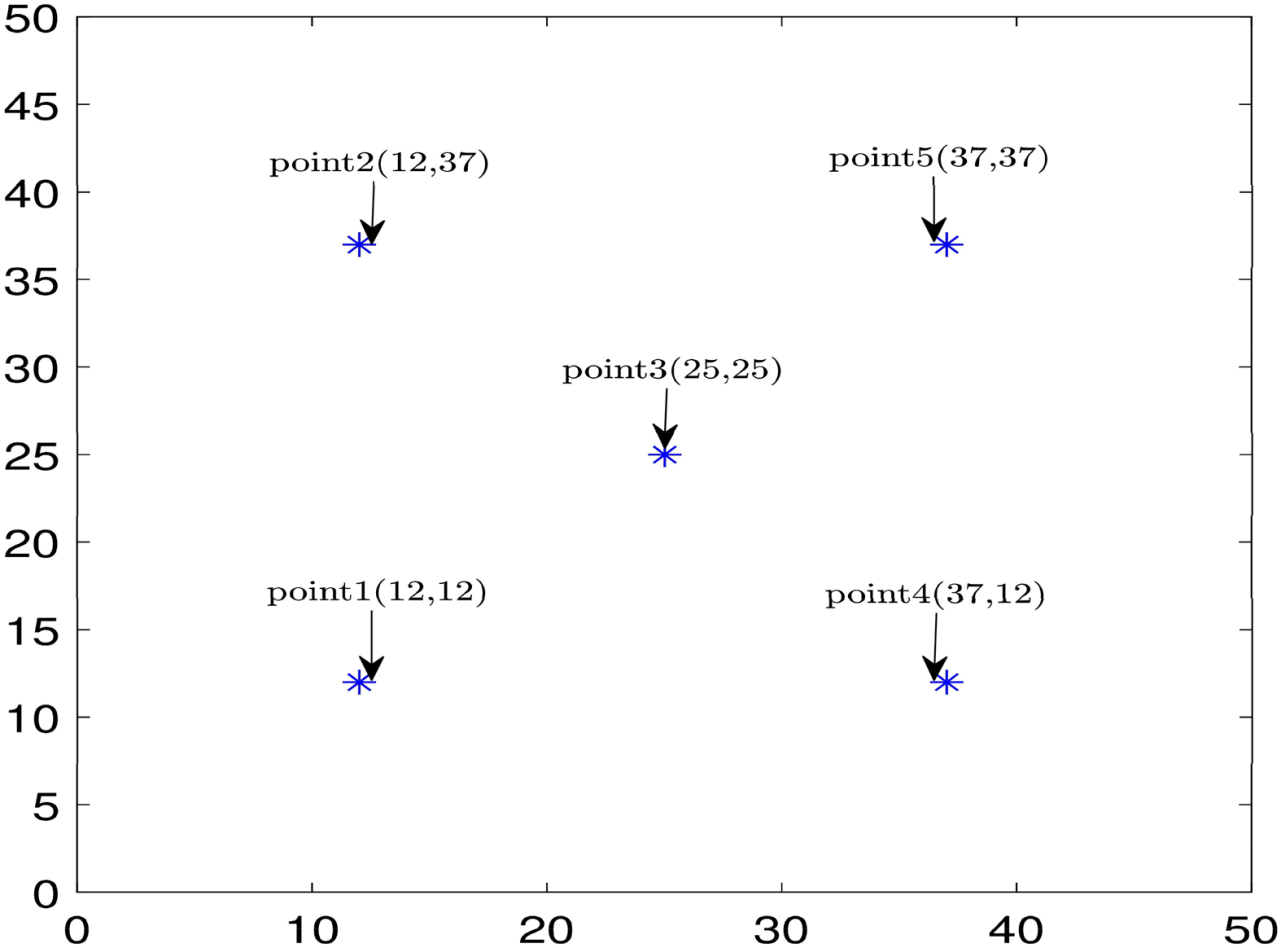
Five selected points in the cavity to evaluate velocity at different times.

**Figure 12:**
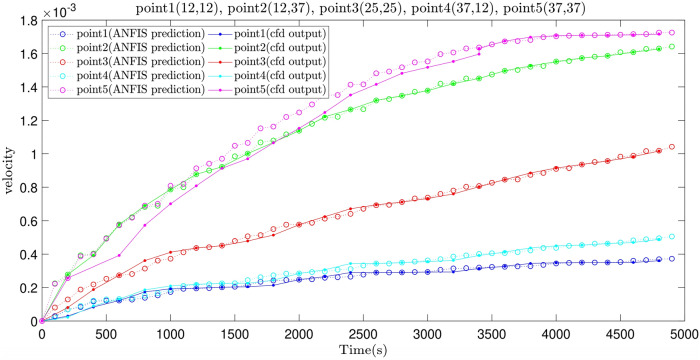
Prediction of the velocity patterns at different times.

Furthermore, in Fig. 10, the velocity prediction patterns for the different times are illustrated. In the following section of this study, we select five points that are highlighted in Fig. 11, and their velocity are predicted at different times, also we compare the predicted velocity of five points with the CFD velocity of five points that are depicted in Fig. 12. Results show that there is a good adaptation between the prediction velocity lines and the CFD velocity lines.

**Table 1:** The equation of MF.

Membership Function	Equation
Bell-shaped	$\frac{1}{{1 + \left| {\frac{x - c}{a}} \right|^{2b} }}$

Generalized Bell-shaped membership function (*Gbellmf*) from different types of MFs, and its equation can be seen in Table 1. *Gbellmf* is selected for prediction of velocity that is obtained from the following equation:

6 $$V = \frac{{\sum\nolimits_{i = 1}^{4} {\sum\nolimits_{j = 1}^{4} {\sum\nolimits_{k = 1}^{4} {\left( {\mu_{1i} \times \mu_{2j} \times \mu_{3k} } \right) \times \left( {p_{m} x + q_{m} y + r_{m} z + s_{m} } \right)} } } }}{{\sum\nolimits_{i = 1}^{4} {\sum\nolimits_{j = 1}^{4} {\sum\nolimits_{k = 1}^{4} {\left( {\mu_{1i} \times \mu_{2j} \times \mu_{3k} } \right)} } } }}$$

$\mu_{1i} , \mu_{1j}$ and $\mu_{1k}$ are written in Eq. 6, as:

7 $$\mu_{1i} = \frac{1}{{\left( {1 + \left| {\frac{{x - c_{i} }}{{a_{i} }} } \right|^{{2b_{i} }} } \right)}}$$

8 $$\mu_{1j} = \frac{1}{{\left( {1 + \left| {\frac{{x - c_{j} }}{{a_{j} }} } \right|^{{2b_{j} }} } \right)}}$$

9 $$\mu_{1k} = \frac{1}{{\left( {1 + \left| {\frac{{x - c_{k} }}{{a_{k} }} } \right|^{{2b_{k} }} } \right)}}$$

Table 2 shows membership function parameters for each input separately in the first layer of ANFIS structure. Also, Table 3 shows Eq. (6) parameters that are extracted from the bottom layer of ANFIS structure, using these parameters in Eq. (6) we can predict fluid velocity based on ANFIS inputs (x and y coordinates and time).

**Table 2:** Membership functions parameters of ANFIS prediction.

Input	MF	Type of MF	*a*	*b*	*c*
X Coordination	in1mf1	Gbellmf	8.35E + 00	5.71E−01	5.97 E−01
	in1mf2	Gbellmf	1.15E + 01	2.30E + 00	1.51E + 01
	in1mf3	Gbellmf	1.22E + 01	3.68E + 00	3.68E + 01
	in1mf4	Gbellmf	7.31E + 00	1.51E + 00	5.24E + 01
Y Coordination	in2mf1	Gbellmf	1.08E + 01	1.63E + 00	3.15E + 00
	in2mf2	Gbellmf	1.58E + 01	3.27E + 00	2.21E + 01
	in2mf3	Gbellmf	9.44E + 00	1.16E + 00	3.88E + 01
	in2mf4	Gbellmf	3.67E + 00	1.44E + 00	5.56E + 01
Time	in3mf1	Gbellmf	8.00E + 02	1.48 E−01	2.66 E−04
	in3mf2	Gbellmf	8.00E + 02	7.82 E−01	1.60E + 03
	in3mf3	Gbellmf	8.00E + 02	1.07E + 00	3.20E + 03
	in3mf4	Gbellmf	8.00E + 02	1.59E + 00	4.80E + 03

**Table 3:** ANFIS prediction parameters in Eq. (6).

Rule	***p***	***q***	***r***	***s***	**Rule**	***p***	***q***	***r***	***s***
1	− 4.69E−06	8.21E−04	2.52E−07	5.15E−03	33	1.40E−05	2.14E−04	1.78E−07	4.75E−03
2	2.39E−04	3.80E−04	− 1.30E−07	6.25E−03	34	− 1.03E−04	− 8.75E−04	− 1.07E−07	− 7.95E−03
3	1.94E−04	− 3.32E−07	− 3.44E−09	4.62E−03	35	− 1.30E−04	4.49E−04	− 1.25E−07	− 1.91E−03
4	1.81E−04	− 1.12E−04	5.33E−08	3.77E−03	36	− 1.35E−04	9.60E−04	− 6.59E−08	1.08E−03
5	8.13E−05	1.13E−03	− 3.84E−07	− 1.69E−02	37	− 5.63E−05	7.81E−04	7.72E−07	− 6.57E−03
6	− 4.34E−04	1.04E−03	7.79E−08	− 1.09E−02	38	1.92E−04	− 1.77E−03	6.86E−08	1.89E−02
7	− 4.29E−04	6.26E−04	− 8.57E−08	− 3.18E−03	39	2.72E−04	− 3.67E−04	1.30E−07	− 8.42E−03
8	− 3.39E−04	4.88E−04	− 9.46E−08	− 7.93E−04	40	2.76E−04	3.50E−04	2.51E−07	− 2.06E−02
9	− 3.02E−04	2.39E−03	9.94E−07	− 1.01E−01	41	1.90E−04	2.35E−03	7.15E−07	− 1.00E−01
10	2.02E−03	2.97E−03	− 8.50E−07	− 1.24E−01	42	− 8.91E−04	− 4.49E−03	3.30E−07	2.05E−01
11	1.49E−03	2.32E−03	− 2.55E−07	− 9.54E−02	43	− 7.94E−04	− 2.32E−03	− 2.90E−08	1.08E−01
12	1.31E−03	2.06E−03	− 7.98E−08	− 8.48E−02	44	− 7.80E−04	− 9.76E−04	− 3.29E−07	5.02E−02
13	3.41E−03	2.52E−02	− 2.18E−06	− 1.30E + 00	45	− 1.38E−03	3.31E−02	7.27E−06	− 1.67E + 00
14	5.05E−03	3.46E−02	− 1.96E−06	− 1.78E + 00	46	− 1.63E−03	− 4.00E−02	− 4.33E−06	2.13E + 00
15	1.77E−03	3.52E−02	− 7.47E−07	− 1.80E + 00	47	− 3.92E−04	− 3.22E−02	− 2.86E−06	1.69E + 00
16	3.85E− 03	3.31E−02	− 4.81E−06	− 1.67E + 00	48	− 6.51E−04	− 2.51E−02	− 4.40E−07	1.31E + 00
17	1.23E−06	8.36E−05	2.02E−07	4.49E−03	49	8.18E−05	4.65E−04	3.04E−07	− 4.06E−04
18	− 2.76E−05	− 8.06E−04	− 8.84E−08	− 1.07E−02	50	− 6.10E−04	5.26E−04	− 3.38E−08	3.60E−02
19	− 2.60E−05	2.89E−05	− 1.50E−07	− 6.85E−03	51	− 7.73E−04	− 3.36E−05	1.08E−07	4.25E−02
20	− 1.80E−05	2.25E−04	− 1.42E−07	− 5.66E−03	52	− 8.57E−04	− 4.16E−04	3.10E−07	4.37E−02
21	− 2.20E−05	6.17E−04	7.06E−07	− 5.45E−03	53	− 3.67E−04	7.37E−04	− 4.40E−07	8.07E−03
22	6.55E−05	− 1.73E−03	1.02E−09	2.28E−02	54	1.20E−03	1.04E−03	− 6.42E−08	− 7.22E−02
23	8.77E−05	− 7.95E−04	8.14E−08	6.35E−03	55	1.68E−03	5.28E−04	− 8.52E−08	− 8.70E−02
24	8.16E−05	− 5.19E−04	9.30E−08	1.59E−03	56	1.81E−03	5.98E−05	− 3.19E−07	− 8.44E−02
25	1.04E−04	2.01E−03	8.93E−07	− 8.17E−02	57	1.43E−03	1.64E−03	1.29E−06	− 1.41E−01
26	− 3.95E−04	− 4.51E−03	3.19E−07	1.85E−01	58	− 5.99E−03	2.71E−03	− 3.67E−07	1.86E−01
27	− 3.86E−04	− 2.94E−03	− 1.13E−07	1.19E−01	59	− 5.82E−03	2.10E−03	− 4.15E−07	2.08E−01
28	− 3.37E−04	− 2.41E−03	− 2.12E−08	9.55E−02	60	− 5.98E−03	1.37E−03	2.14E−07	2.47E−01
29	− 5.88E−04	3.15E−02	8.15E−06	− 1.62E + 00	61	− 8.72E−03	2.15E−02	− 1.38E−06	− 6.64E−01
30	− 4.19E−04	− 4.21E−02	− 3.71E−06	2.20E + 00	62	− 1.02E−02	2.58E−02	2.34E−07	− 8.20E−01
31	2.53E−04	− 3.58E−02	− 3.00E−06	1.86E + 00	63	− 2.63E−03	2.46E−02	8.79E−07	− 1.13E + 00
32	− 3.02E−05	− 3.16E−02	1.35E−07	1.63E + 00	64	− 2.91E−03	2.25E−02	− 2.61E−06	− 9.93E−01

**Figure 13:**
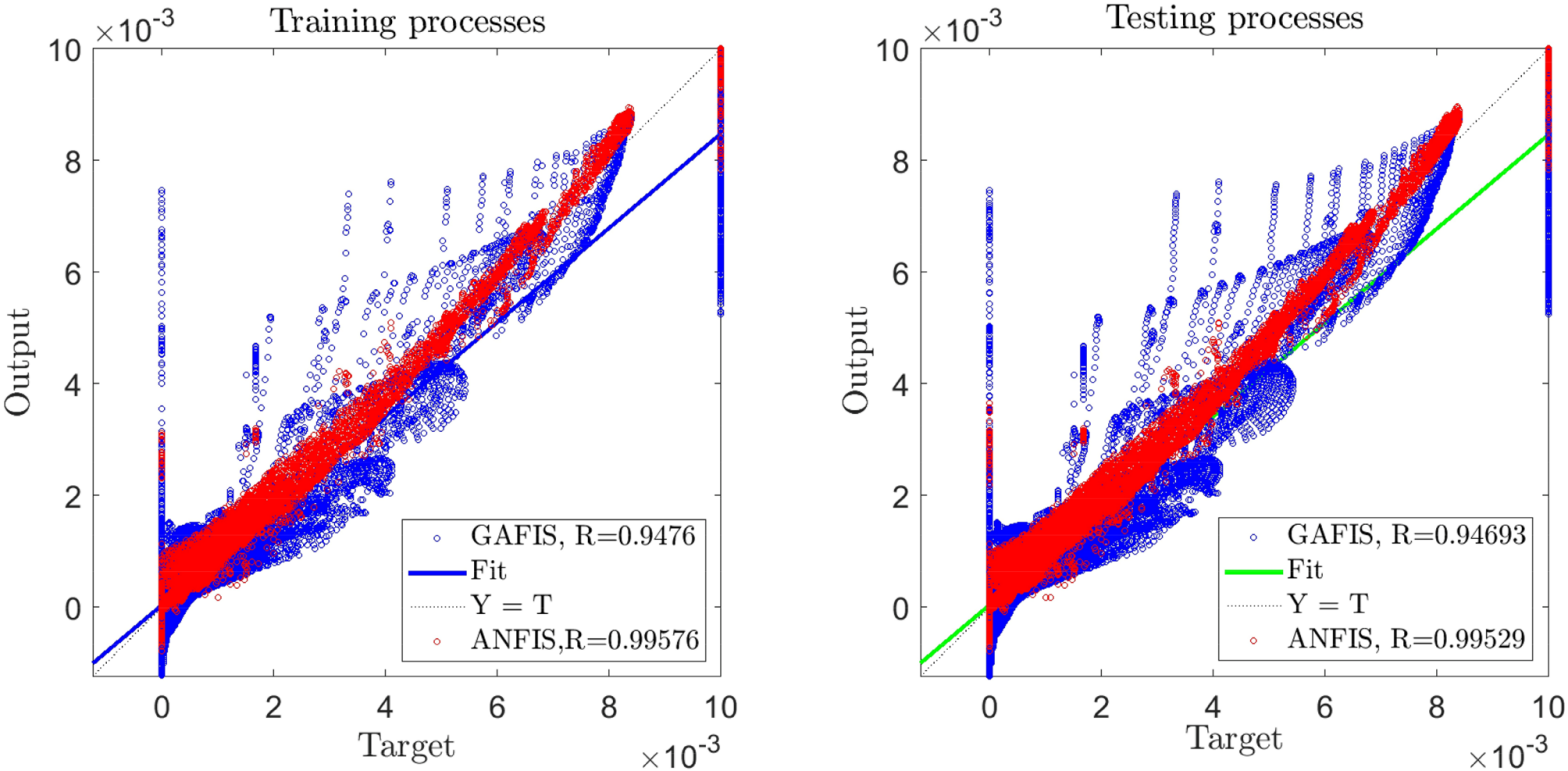
Accuracy comparison of ANFIS and GAFIS methods for the best learning processes.

For better evaluation of the ANFIS method, we compare this method of prediction with the GAFIS method. Similar to the previous analysis, again, we start with training assessment, and after learning data set in both ANFIS and GAFIS, we compare then with R evaluation criteria. The results in Fig. 13 show that the ANFIS method is more capable in the training of CFD dataset, and the ratio of $\frac{{R^{ANFIS} }}{{R^{GAFIS} }} > 1$ that shows the high ability of training data for the ANFIS method. In another assessment, we included more datasets in the process of the assessment called “testing process”. In this stage of evaluation, we observe similar behavior as the training process, and the ANFIS method shows a higher ability with regards to accuracy.

In addition to the accuracy criteria, we compare these methods with regards to error, ${R}^{2}$, STD, and computational time (training and testing times). Table 4 shows that the ANFIS method contains less error than GAFIS when both methods have the same number of iterations, inputs, and percentage of training data set. Both methods reach the best level of accuracy. However, the training time for the ANFIS method is almost two times more than the GAFIS method. In the prediction process, the GAFIS method is even much faster, and the speed of the prediction process is almost 7 times more than the ANFIS method.

**Table 4:** Model parameters, error evaluation, and computational time for ANFIS and GAFIS.

Method	ANFIS	GAFIS
Number of inputs	3	3
Maximum of Iteration	500	500
Percentage of P	60	60
Clustering Type	Grid Partition	FCM Clustering
Training MSE error	2.74166E−08	3.55569E−07
Training RMSE error	0.00016558	0.000596296
Training Mean error	− 5.50537E−11	0.000122548
Training Standard deviation (StD)	0.000165582	0.000583575
Training correlation coefficient (R)	0.995759587	0.94760459
Training coefficient of determination (R^2^)	0.991537156	0.897954459
Testing MSE error	3.06098E−08	3.6226E−07
Testing RMSE error	0.000174957	0.000601881
Testing Mean error	− 5.16564E−07	0.000122067
Testing Standard deviation (StD)	0.000174957	0.000589377
Testing correlation coefficient (R)	0.995291937	0.946933665
Testing coefficient of determination (R^2^)	0.990606039	0.896683366
Learning time(s)	14,189.85973	7129.263288
Prediction time(s)	12.7125845	1.8255114

**Figure 14:**
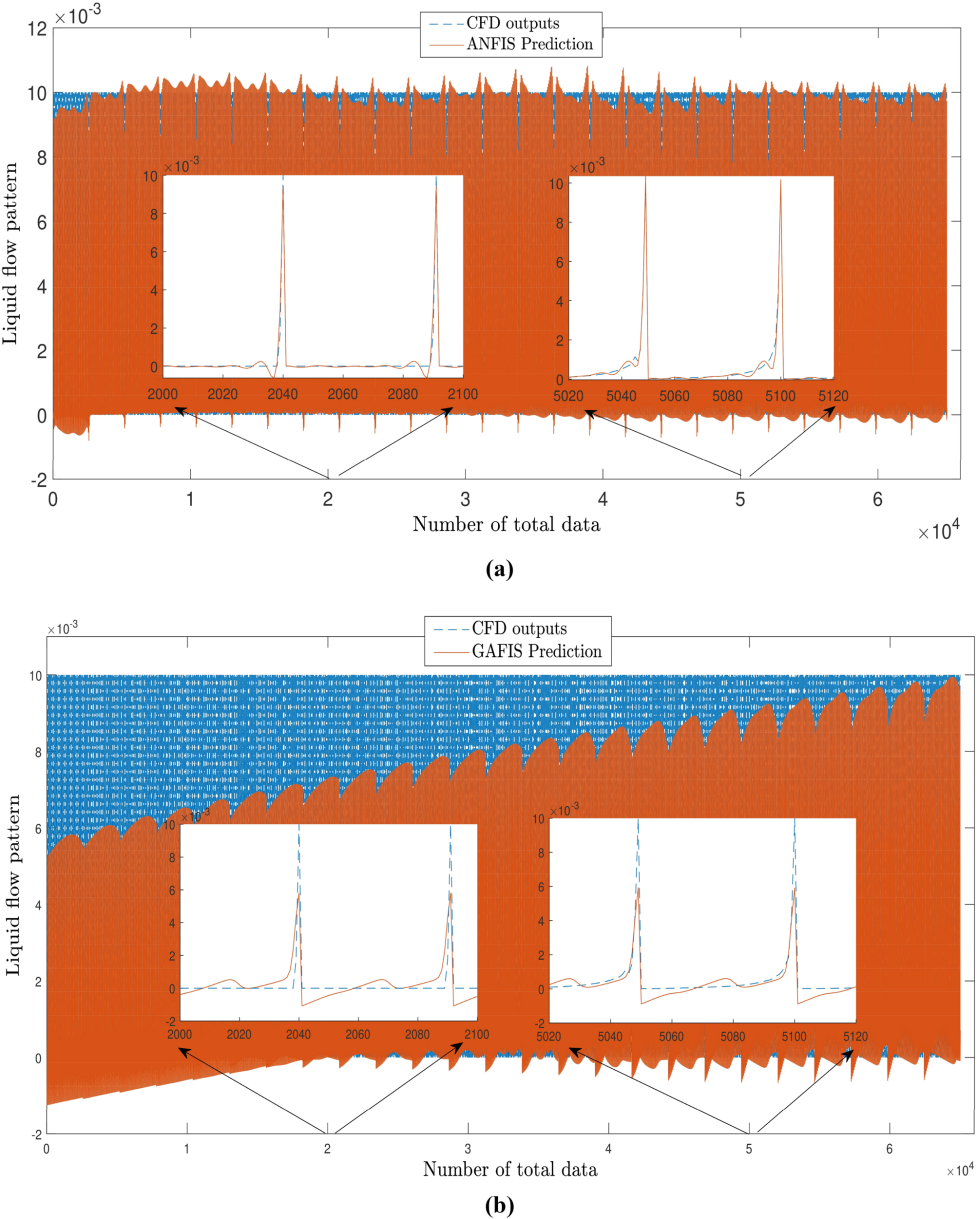
**(a)** Pattern recognition for liquid flow using the ANFIS method. **(b)** Pattern recognition for liquid flow using the GAFIS method.

**Figure 15:**
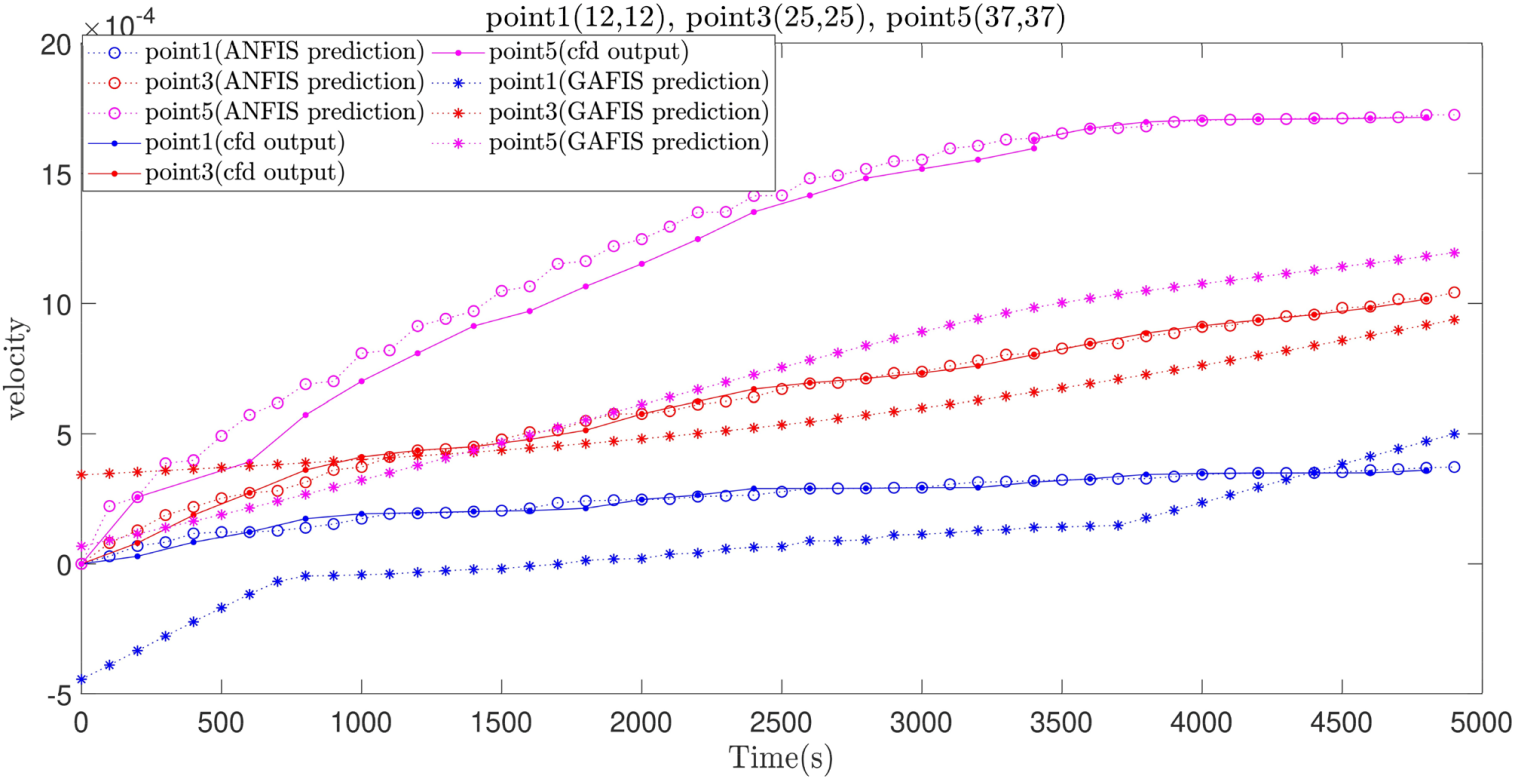
Comparison between ANFIS and GAFIS methods in three different local points in the cavity.

For the better comparison between the ANFIS and GAFIS method, the artificial flow characteristics (velocity distributions) should be compared at local computing points with CFD local dataset. Figure 14a,b shows the flow distribution for the ANFIS and GAFIS method, respectively, and then all velocity distributions are compared with CFD flow distributions in the cavity. The prediction results for the ANFIS method shows that this method can fully predict the flow distribution in the cavity with the minimal difference with CFD dataset, particularly near boundary conditions. However, the prediction results for the GAFIS method show that this method is unable to predict the flow at many local points. The low capability of prediction for some of the local points can be modified with consideration of data filtration near all boundary conditions or introducing boundary conditions as a numerical restriction into the learning algorithms. Another alternative method can be a dense CFD mesh near the boundary condition to have more datasets at those particular locations. We also predict the flow distribution for a different time, and we compare the ANFIS, GAFIS, and CFD methods at the time that the machine learning method does not have training sessions. Figure 15 shows the velocity for ANFIS, GAFIS, and CFD at different points in the cavity domain. Two velocity profiles for ANFIS and GAFIS are completely artificial and based on the prediction ability of machine learning. The results for the ANFIS results are comparable with CFD calculations at different time frames. However, the GAFIS is not as accurate as the ANFIS in the prediction of the flow pattern as a function of time.

For future study, changes in a type of clusterings such as subtractive clustering and fuzzy c-means clustering, and their variable parameters such as the number of cluster in fuzzy c-means clustering and cluster influence range (CIR) in subtractive clustering are worth studying and evaluation.

## Conclusion

In this study, a type of AI called ANFIS method is considered. For ANFIS learning processes, we considered data as inputs and outputs that were extracted from the CFD simulations. In the CFD method, we simulated a cavity and extracted data such as x and y direction and fluid velocity at different times. After making some changes in ANFIS variable parameters to achieve the highest percentage of ANFIS intelligence, ANFIS intelligence became conscious eventually. Fluid velocity as an ANFIS output is predicted in many points of the cavity in comparison with cavity points that are simulated via the CFD method. Particularly, in this study, we considered five points of the cavity at different times and predicted these points at any time in the CFD method period of time. By using this capability of the ANFIS method, we predicted the velocity of the fluid in times that no data existed in the learning processes. For the assessment of prediction capability in the ANFIS method, we compared this method with the GAFIS algorithm. The result shows that the prediction capability in the ANFIS method is higher than the GAFIS method. The ANFIS method can even better predict the flow patter at all local computing points. However, training and prediction time for the ANFIS method is much higher than the GAFIS method.

## References

[1] Shamshirband, S., Babanezhad, M. & Mosavi, A. Prediction of flow characteristics in the bubble column reactor by the artificial pheromone-based communication of biological ants. (2019).

[2] Wang W-C, Chau K-W, Qiu L, Chen Y-B (2015). Improving forecasting accuracy of medium and long-term runoff using artificial neural network based on EEMD decomposition. Environ. Res..

[3] Wu C, Chau K (2011). Rainfall–runoff modeling using artificial neural network coupled with singular spectrum analysis. J. Hydrol..

[4] Babanezhad M, Nakhjiri AT, Shirazian S (2020). Changes in the number of membership functions for predicting the gas volume fraction in two-phase flow using grid partition clustering of the ANFIS method. ACS Omega.

[5] Yilmaz I, Kaynar O (2011). Multiple regression, ANN (RBF, MLP) and ANFIS models for prediction of swell potential of clayey soils. Expert Syst. Appl..

[6] Boyacioglu MA, Avci D (2010). An adaptive network-based fuzzy inference system (ANFIS) for the prediction of stock market return: the case of the Istanbul stock exchange. Expert Syst. Appl..

[7] Nguyen Q, Babanezhad M, Taghvaie Nakhjiri A, Rezakazemi M, Shirazian S (2020). Prediction of thermal distribution and fluid flow in the domain with multi-solid structures using Cubic-Interpolated Pseudo-Particle model. PLoS ONE.

[8] Nguyen Q, Taghvaie Nakhjiri A, Rezakazemi M, Shirazian S (2020). Thermal and flow visualization of a square heat source in a nanofluid material with a cubic-interpolated pseudo-particle. ACS Omega.

[9] Xu P, Babanezhad M, Yarmand H, Marjani A (2019). Flow visualization and analysis of thermal distribution for the nanofluid by the integration of fuzzy c-means clustering ANFIS structure and CFD methods. J. Visualization.

[10] Tian E, Babanezhad M, Rezakazemi M, Shirazian S (2019). Simulation of a bubble-column reactor by three-dimensional cfd: multidimension-and function-adaptive network-based fuzzy inference system. Int J Fuzzy Syst.

[11] Pourtousi M, Sahu J, Ganesan P, Shamshirband S, Redzwan G (2015). A combination of computational fluid dynamics (CFD) and adaptive neuro-fuzzy system (ANFIS) for prediction of the bubble column hydrodynamics. Powder Technol..

[12] Selimefendigil F, Öztop HF (2018). Numerical analysis and ANFIS modeling for mixed convection of CNT-water nanofluid filled branching channel with an annulus and a rotating inner surface at the junction. Int. J. Heat Mass Transf..

[13] Jawad HL, Abdullah S, Zulkifli R, Mahmood W (2012). Prediction of centrifugal compressor performance by using adaptive neuro-fuzzy inference system (ANFIS). IREMOS.

[14] Rezakazemi M, Shirazian S (2019). Gas-liquid phase recirculation in bubble column reactors: development of a hybrid model based on local CFD–adaptive neuro-fuzzy inference system (ANFIS). J. Non-Equilib. Thermodyn..

[15] Nabipour N, Babanezhad M, Taghvaie Nakhjiri A, Shirazian S (2020). Prediction of nanofluid temperature inside the cavity by integration of grid partition clustering categorization of a learning structure with the fuzzy system. ACS Omega.

[16] Pourtousi M, Zeinali M, Ganesan P, Sahu JN (2015). Prediction of multiphase flow pattern inside a 3D bubble column reactor using a combination of CFD and ANFIS. RSC Adv..

[17] Pourtousi M (2016). CFD modelling and anfis development for the hydrodynamics prediction of bubble column reactor ring sparger.

[18] Mitra, P., Maulik, S., Chowdhury, S. & Chowdhury, S. in 2007 42nd International Universities Power Engineering Conference. 397–401 (IEEE).

[19] Yun Z (2008). RBF neural network and ANFIS-based short-term load forecasting approach in real-time price environment. IEEE Trans. Power Syst..

[20] Abdulshahed AM, Longstaff AP, Fletcher S (2015). The application of ANFIS prediction models for thermal error compensation on CNC machine tools. Appl. Soft Comput..

[21] Azwadi CSN, Zeinali M, Safdari A, Kazemi A (2013). Adaptive-network-based fuzzy inference system analysis to predict the temperature and flow fields in a lid-driven cavity. Numer. Heat Transfer, Part A Appl..

[22] Takagi T, Sugeno M (1985). Fuzzy identification of systems and its applications to modeling and control. IEEE Trans. Syst. Man Cybern..

